# Supplementary material for: Amino acid insertion in Bat MHC-I enhances complex stability and augments peptide presentation
Source: Commun Biol. 2024 May 16;7:586. doi: 10.1038/s42003-024-06292-5 (PMC11099071; doi:10.1038/s42003-024-06292-5)
Supplement: Supplementary file 2 — Supplementary Information [file 42003_2024_6292_MOESM2_ESM.pdf]

Supplementary Figure 1. Sequence characterization of the AA insertions in bat MHC-Is of Mylu-B*67:01

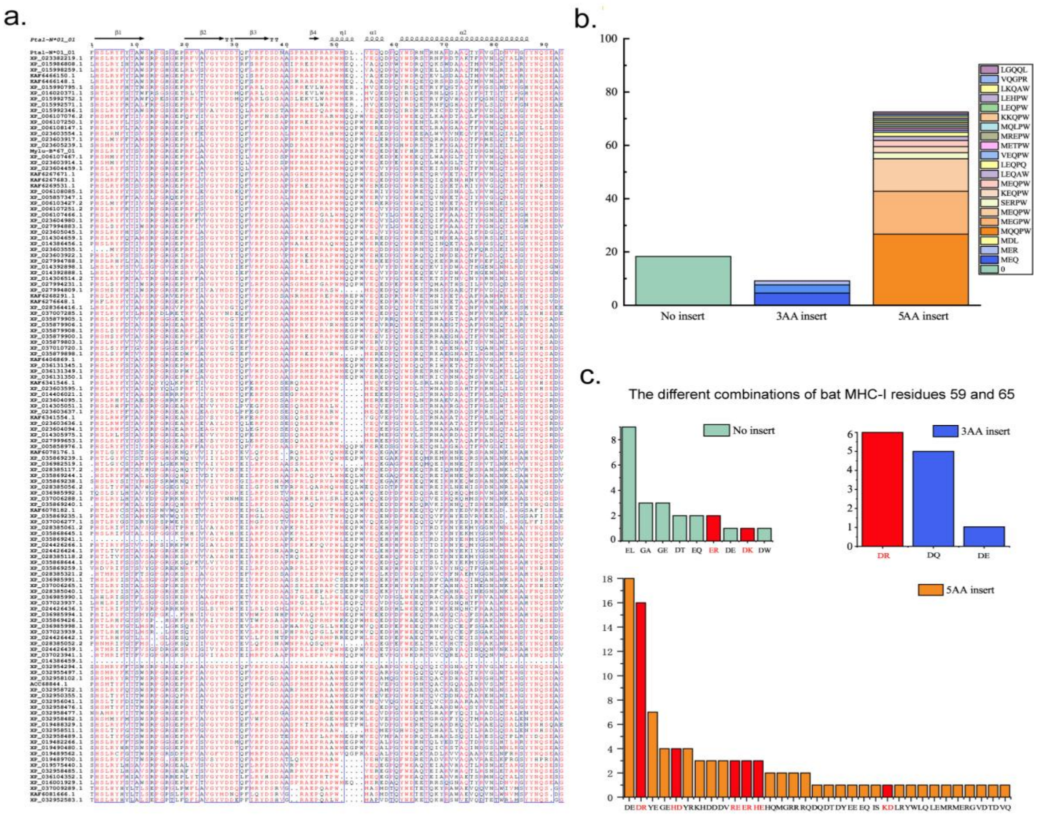

(a) Sequence comparison of the α1 region of the bat MHC class I molecules.

(b) Statistics of different insertion sequences in bat MHC-Is. Different insertions correspond to different colors.

(c) Statistics of positive and negative charge residue pairing after insertion. Pairs are highlighted in red. The positions of paired residues in 3AA are 59th and 65th, and in 5AA insertion are 61th and 67th.The pairing of this residue combination (D61 and R67) accounts for about 17% of the 5AA insertion sequence, and the pairing of residues with the potential to form a salt bridge accounts for 31%.

Supplementary Figure 2. Gel filtration chromatogram of Mylu-B*67:01 and Mylu-B*67:01ΔMQQPW refolded in vitro with three COVID-19th peptides (P1, P2 and P3). All three peptides were screened from the spike protein based on the PBM of Mylu-B*67:01.

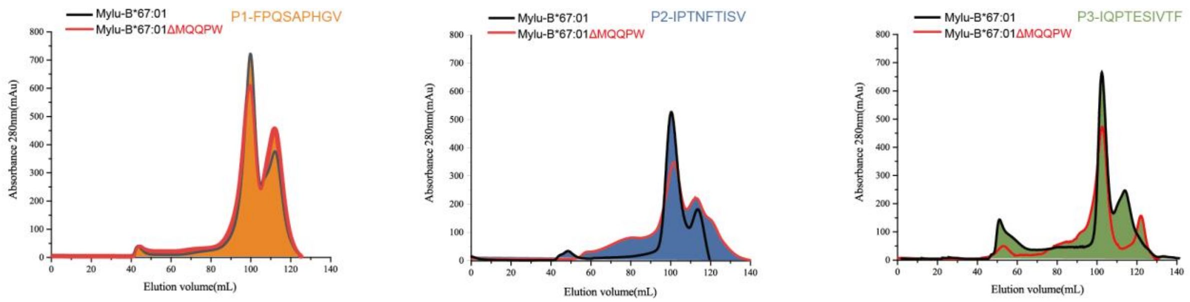

Supplementary Figure 3. Comparison between the Mylu-B*67:01 structure predicted by swiss-Model and the resolved structure. The MQQPW insertion was marked with red circles.

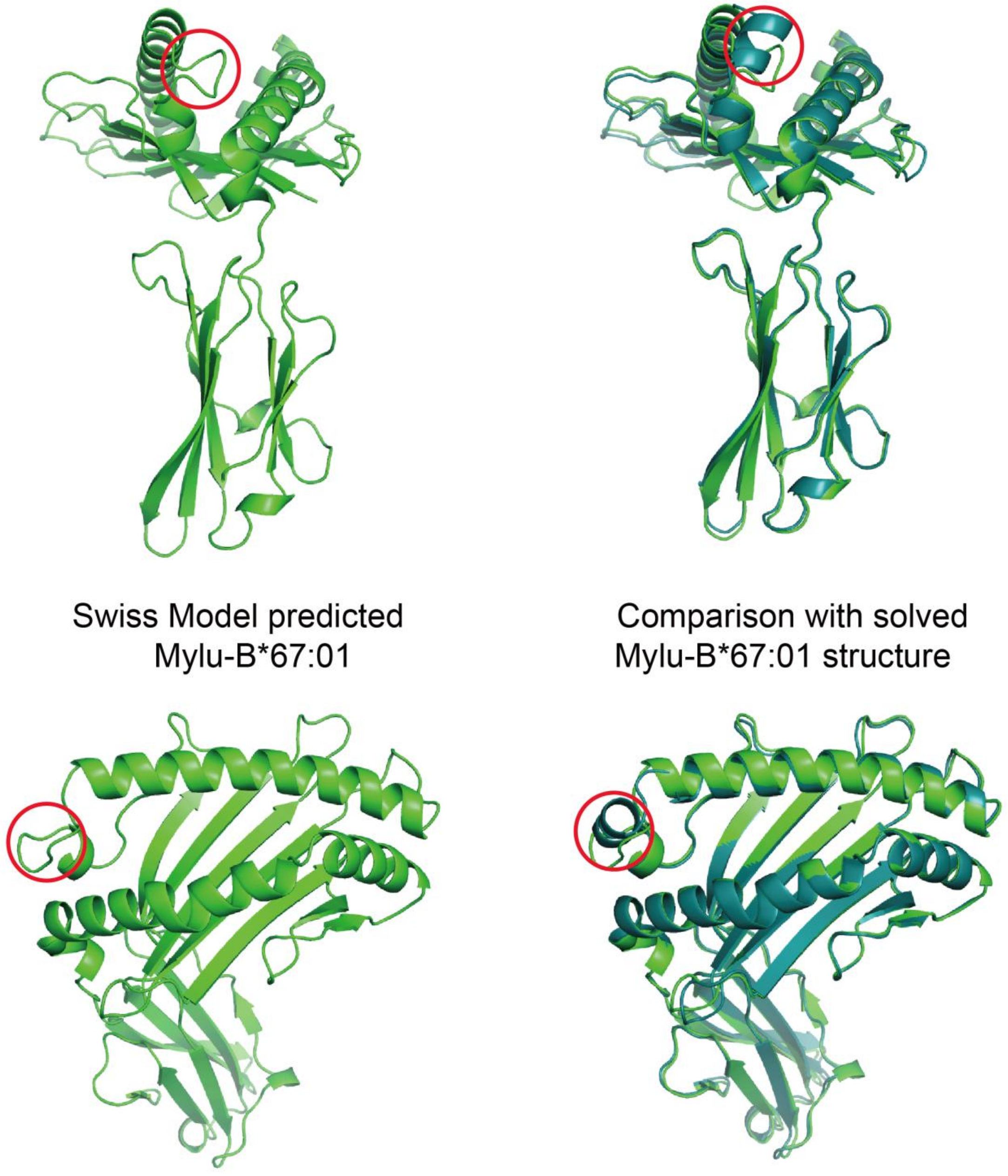

Supplementary Figure 4. Sequence alignment and AF2 prediction of four bat MHC I sequences without the W58 in the insertion.

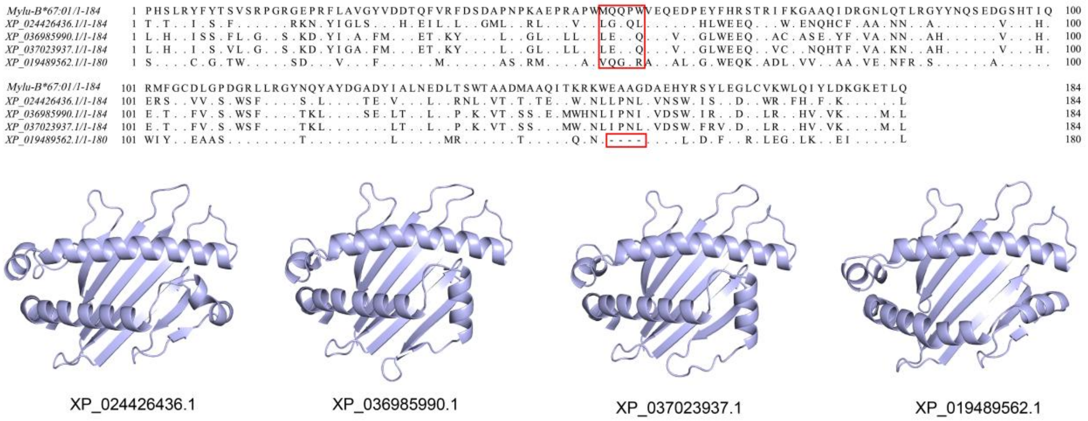

Supplementary Table 1. Bat MHC I molecular sequence information

Supplementary Table 1.Bat MHC I molecular sequence information

Species	Version	Insert	61AA	67AA	Saltbridge Network
Mylu-B-67:01	XP_006107180.2	5	D	R	YES
Myotis_lucifugus	XP_014305051	0	E	R	NO
	XP_014305975	0	E	L	NO
	XP_023603595	0	E	L	NO
	XP_023603636	0	E	L	NO
	XP_023603637	0	E	L	NO
	XP_023604094	0	E	L	NO
	XP_023604095	0	E	L	NO
	XP_006103427	5	R	G	NO
	XP_006107076	5	D	E	NO
	XP_006107250	5	D	E	NO
	XP_006107251	5	Y	R	NO
	XP_006107466	5	Y	E	NO
	XP_006107467	5	D	E	NO
	XP_006108085	5	Y	R	NO
	XP_006108147	5	D	E	NO
	XP_014304659	5	D	R	YES
	XP_014306514	5	Y	E	YES
	XP_014386459	5	R	E	YES
	XP_023603554	5	D	E	NO
	XP_023603555	5	D	R	YES
	XP_023603914	5	D	E	NO
	XP_023603917	5	R	R	NO
	XP_023603922	5	D	R	YES
	XP_023604459	5	Y	R	NO
	XP_023604980	5	Y	E	NO
	XP_023605045	5	Y	R	NO
	XP_023605239	5	R	R	NO
Phyllostomus_discolor	KAF6081666	0	D	T	NO
	XP_028385052	0	G	E	NO
	XP_035879898	0	D	E	NO
	KAF6078176	5	G	E	NO
	KAF6078182	5	D	E	NO
	XP_028364416	5	D	T	NO
	XP_028385040	5	K	H	NO
	XP_028385056	5	D	E	NO
	XP_028385061	5	H	D	YES
	XP_028385117	5	D	E	NO
	XP_028385118	5	H	D	YES

	XP_028385321	5	K	D	YES
	XP_035868644	5	D	Y	NO
	XP_035868645	5	H	E	YES
	XP_035869235	5	N	E	YES
	XP_035869238	5	G	E	NO
	XP_035869239	5	G	E	NO
	XP_035869240	5	D	D	NO
	XP_035869241	5	H	D	YES
	XP_035869244	5	G	E	NO
	XP_035869259	5	H	E	YES
	XP_035869426	5	D	E	NO
	XP_035879803	5	Y	E	NO
	XP_035879900	5	I	S	NO
	XP_035879905	5	R	E	YES
	XP_035879906	5	R	E	YES
	XP_035879908	5	E	E	NO
Artibeus_jamaicensis	XP_036982519	0	G	E	NO
	XP_037009289	0	D	T	NO
	XP_036985990	5	D	E	NO
	XP_036985991	5	K	H	NO
	XP_036985992	5	D	D	NO
	XP_036985994	5	D	E	NO
	XP_036985998	5	D	E	NO
	XP_037006265	5	K	H	NO
	XP_037006277	5	D	Q	NO
	XP_037006288	5	D	D	NO
	XP_037007285	5	D	V	NO
	XP_037010720	5	D	R	YES
	XP_037023937	5	D	E	NO
	XP_037023939	5	D	E	NO
	XP_037023941	5	V	D	NO
Rhinolophus_ferrumequinum	XP_032952583	0	D	K	NO
	XP_032958485	0	G	A	NO
	XP_032958511	0	E	Q	NO
	ACC68844	5	E	R	YES
	XP_032950355	5	E	R	YES
	XP_032954294	5	R	Q	YES
	XP_032955497	5	R	Q	YES
	XP_032956041	5	V	Q	NO
	XP_032958102	5	M	G	NO
	XP_032958476	5	D	E	NO
	XP_032958477	5	D	R	YES
	XP_032958482	5	E	Q	NO
	XP_032958489	5	M	R	NO

	XP_032958722	5	M	G	NO
Rousettus_aegyptiacus	XP_015990795	3	D	Q	NO
	KAF6466148	3	D	Q	NO
	XP_015986808	3	D	R	YES
	KAF6466150	3	D	R	YES
	XP_015992346	3	D	R	YES
	XP_015992571	3	D	Q	NO
	XP_015992752	3	D	Q	NO
	XP_015998259	3	D	R	YES
	XP_016001929	3	D	E	NO
	XP_016020371	3	D	Q	NO
Myotis_brandtii	XP_014406021	0	E	Q	NO
	XP_005857347	5	Y	W	NO
	XP_005858976	5	D	R	YES
	XP_014386456	5	D	R	YES
	XP_014392888	5	Y	E	NO
	XP_014392898	5	Y	E	NO
Desmodus_rotundus	XP_024426266	5	H	D	NO
	XP_024426424	5	H	Q	NO
	XP_024426436	5	D	E	NO
	XP_024426439	5	T	D	NO
	XP_024426442	5	D	E	NO
Eptesicus_fuscus	XP_027994809	0	D	W	NO
	XP_027999653	0	E	L	NO
	XP_027994231	5	D	R	YES
	XP_027994788	5	E	R	YES
	XP_027994883	5	Y	E	NO
Hipposideros_armiger	XP_019489700	0	E	R	YES
	XP_019482266	5	L	R	NO
	XP_019488329	5	D	R	YES
	XP_019489562	5	L	Q	NO
	XP_019490480	5	L	E	NO
Molossus_molossus	XP_036104352	0	G	A	NO
	KAF6406869	5	D	R	YES
	XP_036131345	5	H	Q	NO
	XP_036131349	5	D	R	YES
	XP_036131350	5	D	R	YES
Myotis_myotis	KAF6341546	0	E	L	NO
	KAF6341554	0	E	L	NO
	KAF6269531	5	D	R	YES
	KAF6276648	5	D	V	NO
Pipistrellus_kuhlii	KAF6267671	5	D	R	YES
	KAF6267683	5	D	R	YES

	KAF6268291	5	D	V	NO
Pteropus alecto	KT987929	3	D	R	YES
Pteropus_vampyrus	XP_023382219	3	D	R	YES
Rhinolophus_sinicus	XP_019575440	0	G	A	NO

The salt bridge network refers to the ability to form a salt bridge network between amino acids 61 and 67 and the P1 position of the polypeptide in the structure predicted by AF2.
61AA and 67AA are based on the amino acid sequence of the sequence MYLU-B-67.

Supplementary Table 2. Peptide Information

Supplementary Table 2.Peptide Information

peptide name	sequence	Origin
P1/P1-F	FPQSAPHGV	SARS-CoV-2 spike protein
P2	IPTNFTISV	SARS-CoV-2 spike protein
P3	QPTESIVTF	SARS-CoV-2 spike protein
P1-T	TPQSAPHGV	Variant of P1
P1-E	EPTSAPHGV	Variant of P1
Ran_7Xsplitted	XXXXXXX	
Ran_8Xsplitted	XXXXXXXX	
Ran_9Xsplitted	XXXXXXXXX	
Ran_10Xsplitted	XXXXXXXXXX	
Ran_11Xsplitted	XXXXXXXXXXX	
Ran_12Xsplitted	XXXXXXXXXXXX	
Ran_13Xsplitted	XXXXXXXXXXXXX	
Ran_14Xsplitted	XXXXXXXXXXXXXX	
Ran_15Xsplitted	XXXXXXXXXXXXXXX	
Ran_16Xsplitted	XXXXXXXXXXXXXXXX	
Ran_17Xsplitted	XXXXXXXXXXXXXXXXX	

P1,P2,P3 were obtained by screening the spike protein of SARS-CoV-2 based on the anchor site of the MYLU-B-67 protein binding motif.
P1-E,P1-T are mutations in the first F of P1 based on the T/E preference of the first position of the protein binding motif of MYLU-B-67ΔMQQPW.
Ran_7Xsplitted-Ran_17Xsplitted are synthetic random peptide libraries.where X is a random amino acid other than cysteine
